# Bone Turnover Markers and Probable Advanced Nonalcoholic Fatty Liver Disease in Middle-Aged and Elderly Men and Postmenopausal Women With Type 2 Diabetes

**DOI:** 10.3389/fendo.2019.00926

**Published:** 2020-01-28

**Authors:** Ningjian Wang, Yuying Wang, Xiaoman Chen, Wen Zhang, Yi Chen, Fangzhen Xia, Heng Wan, Qing Li, Boren Jiang, Bin Hu, Yingli Lu

**Affiliations:** ^1^Institute and Department of Endocrinology and Metabolism, Shanghai Ninth People's Hospital, Shanghai JiaoTong University School of Medicine, Shanghai, China; ^2^Department of Prosthodontics, Shanghai Ninth People's Hospital, Shanghai JiaoTong University School of Medicine, Shanghai, China

**Keywords:** type 2 diabetes mellitus, nonalcoholic fatty liver disease, advanced fibrosis, nonalcoholic steatohepatitis, bone turnover markers

## Abstract

**Objective:** Type 2 diabetic patients have a higher incidence of nonalcoholic steatohepatitis (NASH) and advanced stages of fibrosis, and nonalcoholic fatty liver disease (NAFLD) is associated with impaired bone health. We aimed to investigate whether bone turnover is associated with the probable presence of NASH and fibrosis.

**Methods:** In total, 4,937 diabetic participants from Shanghai, China were enrolled in 2018. Subjects with NAFLD were categorized into simple NAFLD and probable NASH groups based on the presence of a metabolic syndrome. The NAFLD fibrosis score was used to identify patients with a higher likelihood of advanced fibrosis.

**Results:** In postmenopausal women, large N-mid fragment of osteocalcin (N-MID osteocalcin) was negatively associated with probable NASH (*P for trend* < 0.001). β*-*C-terminal cross-linked telopeptides of type I collagen (β-CTX) and procollagen type I N-terminal propeptide (P1NP) were positively associated with the probable presence of significant fibrosis in postmenopausal women (*P for trend* 0.015 and <0.001). However, in men, N-MID osteocalcin and β-CTX were negatively associated with the probable presence of significant fibrosis (*P for trend* 0.029 and 0.027).

**Conclusions:** Significant associations among N-MID osteocalcin, β-CTX and P1NP, and probable advanced NAFLD were observed. Further prospective and animal studies are warranted to understand the causal relationship and underlying mechanism.

## Introduction

Nonalcoholic fatty liver disease (NAFLD) encompasses a histological spectrum from nonalcoholic fatty liver and nonalcoholic steatohepatitis (NASH) to fibrosis and cirrhosis and can even develop into hepatocellular carcinoma (1). Type 2 diabetic patients not only have a much higher prevalence of NAFLD (2) but also a higher prevalence of NASH and advanced stages of fibrosis (3, 4). NASH has now become the second leading cause of liver transplantation in the United States (5), and fibrosis is the most important histological feature of NAFLD associated with long-term mortality (6). Therefore, intensive attention should be paid to the advanced form of NAFLD, especially in diabetic patients.

Altered bone turnover is associated with NAFLD, but there is only scarce evidence. NAFLD is associated with a self-reported history of osteoporotic fractures in middle-aged and elderly individuals (7, 8). Moreover, a relatively small sample study in postmenopausal women with diabetes found that the presence of NAFLD and significant fibrosis was significantly associated with lower bone turnover (9). Bone turnover markers, such as β-C-terminal telopeptide (β-CTX), N-MID osteocalcin, and procollagen type 1 N-peptide (P1NP), were reported to be associated with chronic liver injury, inflammation, and fibrosis (10–12). However, to the best of our knowledge, there are limited studies with a large sample size that have provided information on the association between advanced NAFLD status (NASH and significant fibrosis) and bone turnover markers in diabetic patients of both genders.

Thus, we hypothesize that bone metabolism may be associated with the process of NAFLD. Because liver biopsies could not be applied to a large population, we used metabolic syndrome (MetS), a strong noninvasive NASH predictor (13), to assess NASH in this study. The NAFLD fibrosis score (NFS) is a noninvasive system that identifies liver fibrosis in patients with NAFLD (14). Angulo et al. describe it as a scoring system that accurately separates patients with NAFLD with and without advanced fibrosis (14). In this large community-based study, we aimed to investigate whether bone turnover markers (β-CTX, N-MID osteocalcin, and P1NP) are associated with the probable presence of NASH and significant fibrosis evaluated using the above noninvasive measurements in Chinese men and postmenopausal women with type 2 diabetes.

## Materials and Methods

### Study Design and Participants

A population-based study named the METAL study (Environmental Pollutant Exposure and Metabolic Diseases in Shanghai, ChiCTR1800017573, www.chictr.org.cn) was conducted in 2018 (15). Participants were randomly enrolled from seven communities in Huangpu and Pudong District, Shanghai, China and were randomly selected from half of the type 2 diabetic patients in the registration platform of each community healthcare center. Chinese citizens at least 18 years of age who had lived in their current area for 6 months were included. In total, 4,937 subjects with diabetes who were 23–99 years old received an examination. We excluded participants who had missing laboratory results (*n* = 8) and questionnaire data (*n* = 116), had a history of excessive consumption (male ≥ 140 g/week, female ≥ 70 g/week) of pure alcohol (Chinese Society of Hepatology 2010) (*n* = 225), had self-reported viral hepatitis (including hepatitis B and hepatitis C viruses) (*n* = 243), had used medications associated with secondary NAFLD (corticosteroids, estrogens, amiodarone, methotrexate) (*n* = 157), or had no ultrasound (*n* = 68) or bone turnover marker (*n* = 40) results. Then, we excluded 86 premenopausal women and 12 men less than 40 years of age. Finally, 1,764 men and 2,218 postmenopausal women were included (Supplementary Figure 1).

The study protocol was approved by the Ethics Committee of Shanghai Ninth People's Hospital, Shanghai Jiao Tong University School of Medicine. The study protocol conformed to the ethical guidelines of the 1975 Declaration of Helsinki as reflected in the *a priori* approval by the appropriate institutional review committee. Informed consent was obtained from all participants included in the study.

### Measurements

We used a questionnaire that assessed sociodemographic characteristics, medical history, family history, and lifestyle factors. The same group of trained and experienced personnel in the SPECT-China study (16, 17) conducted the interviews and clinical examinations according to a standard protocol. Current smoking was defined as having smoked at least 100 cigarettes in one's lifetime and is currently smoking cigarettes (18). Waist circumference was measured in the horizontal plane midway between the lowest ribs and the iliac crest as suggested by the World Health Organization and the International Diabetes Federation (19).

Blood samples were obtained between 6:00 and 9:00 a.m. after overnight fasting for at least 8 h. Blood was refrigerated immediately after phlebotomy; after 2 h, it was centrifuged, and the serum was aliquoted and frozen in a central laboratory. Glycated hemoglobin (HbA1c) was measured *via* high-performance liquid chromatography (MQ-2000PT, Medconn, Shanghai, China). Fasting plasma glucose, alanine aminotransferase (ALT), aspartate aminotransferase (AST), and lipid profiles were performed with a Beckman Coulter AU 680 (Brea, USA). β-C-terminal telopeptide (β-CTX), N-MID osteocalcin, and procollagen type 1 N-peptide (P1NP) were detected with a chemiluminescence method (Roche E602, Switzerland). The interassay coefficients of variation were as follows: 3.30% (P1NP), 1.81% (N-MID osteocalcin), and 7.60% (β-CTX). The intra-assay coefficients of variation were as follows: 3.0% (P1NP), 0.80% (N-MID osteocalcin), and 5.50% (β-CTX).

Hypertension was defined as systolic blood pressure ≥140 mmHg, diastolic blood pressure ≥90 mmHg, or self-reported previous diagnosis of hypertension by physicians. Dyslipidemia was defined as total cholesterol ≥6.22 mmol/L (240 mg/dl), triglycerides ≥2.26 mmol/L (200 mg/dl), LDL-C ≥ 4.14 mmol/L (160 mg/dl), HDL-C <1.04 mmol/L (40 mg/dl), or self-reported previous diagnosis of hyperlipidemia by physicians, according to the modified National Cholesterol Education Program-Adult Treatment Panel III.

### Variable Definition

Liver fat accumulation (steatosis) was detected by ultrasound (Mindray M7, MINDRAY, Shenzhen, China) (20, 21). According to the criteria proposed by Saadeh et al., presentation of steatosis included increased liver echogenicity, stronger echoes in the hepatic parenchyma compared to the renal parenchyma, vessel blurring, and narrowing of the lumen of the hepatic veins (22).

According to the guidelines of the American Association for the Study of Liver Disease (AASLD) and Chinese Society of Hepatology, the presence of MetS is a strong predictor of the presence of steatohepatitis in patients with NAFLD (one of the guidance statements from the AASLD) (23). Thus, we categorized the subjects with MetS and NAFLD into subjects with probable NASH and the remaining subjects as those with simple NAFLD. MetS was determined based on the International Diabetes Federation criteria (2005) (24).

NAFLD fibrosis score (NFS) was used to identify NAFLD patients with a higher likelihood of having bridging fibrosis (stage 3) or cirrhosis (stage 4), which was also suggested by the AASLD. The following formula was used to calculate the NFS: −1.675 + 0.037 ^*^ age (years) + 0.094 ^*^ body mass index (kg/m^2^) + 1.13 ^*^ IFG/diabetes (yes = 1, no = 0) + 0.99 ^*^ AST/ALT ratio −0.013 ^*^ platelet (^*^10^9^/l) −0.66 ^*^ albumin (g/dl) (14). A score < −1.455 indicated the likely absence of significant fibrosis and had 90% sensitivity and 60% specificity to exclude advanced fibrosis, whereas a score >0.676 indicated the likely presence of significant fibrosis and had 67% sensitivity and 97% specificity to identify the presence of advanced fibrosis (23). NAFLD subjects with scores between −1.455 and 0.676 were considered to have indeterminate results.

The estimated glomerular filtration rate (eGFR) was calculated according to the Chronic Kidney Disease Epidemiology Collaboration (CKD-EPI) equation for “Asian origin” (25).

### Statistical Analysis

Data analyses were performed using IBM SPSS Statistics software, Version 22 (IBM Corporation, Armonk, NY, USA). A (two-sided) *P* <0.05 indicated significance. Continuous variables were summarized as the mean ± SD and categorical variables as percentages (%). Linear or logistic regression analysis was used to test for trends in variables associated with inflammatory NAFLD progression (from non-NAFLD to simple NAFLD to probable NASH) and fibrotic progression (from absence of significant fibrosis to having indeterminate results to the presence of significant fibrosis). The concentrations of P1NP, N-MID osteocalcin and β-CTX were logarithmically transformed to achieve a normal distribution if needed in the analyses.

P1NP, N-MID osteocalcin, and β-CTX were divided into quartiles, with the first quartile representing the lowest level and the fourth quartile the highest level. Multinomial logistic regression was used to measure the association between bone turnover markers (independent variable) and simple NAFLD and probable NASH (dependent variable), adjusting for age, duration of diabetes, HbA1c, current smoking, waist circumference, eGFR, dyslipidemia, hypertension, and use of metformin or thiazolidinediones. Multinomial logistic regression was also used to analyze their association with the indeterminate results and advanced fibrosis (dependent variable) using the same model.

## Results

### Characteristics of the Diabetic Participants Categorized by NAFLD Progression

This study included 3,982 diabetic participants with a mean age of 68 years old (SD 8, max 99, min 41). Overall, 44.3% of the participants were men. The median P1NP, N-MID osteocalcin, and β-CTX levels were 34.4 ng/ml (IQR 27.1–43.8), 9.5 ng/ml (IQR 7.5–11.8), and 0.17 ng/ml (IQR 0.12–0.23), respectively, in men and 44.4 ng/ml (IQR 34.8–57.7), 12.3 pg/ml (IQR 9.7–15.7), and 0.22 ng/ml (IQR 0.16–0.29), respectively, in postmenopausal women.

From non-NAFLD to simple NAFLD and probable NASH, subjects were relatively younger and had a worse metabolic profile (greater obesity indices, HbA1c, blood pressure, and a worse lipid profile) (Table 1). However, from probable absence, indetermination, and probable presence of significant fibrosis, subjects were significantly older and had a longer duration of diabetes and inconsistent metabolic profile changes (greater waist circumference and blood pressure but better lipid profile and glycemic indices) (Table 2).

**Table 1:** Characteristics of the study participants sorted into NAFLD categories.

**Characteristic**	**Non-NAFLD**	**Simple NAFLD**	**Probable NASH**	***P* for trend**
**MEN**				
*N*	566	336	862	
Age, year	69.7 ± 8.3	66.8 ± 8.3	67.7 ± 8.4	<0.001
Duration of diabetes, year	12.0 ± 8.2	9.5 ± 6.8	9.5 ± 7.3	<0.001
Current smoking, %	33.0	34.2	32.7	0.864
AST/ALT	1.24 ± 0.39	1.10 ± 0.37	1.06 ± 0.43	<0.001
Waist circumference, cm	86.7 ± 7.6	86.1 ± 5.2	98.2 ± 6.9	<0.001
FPG, mmol/L	7.7 ± 2.4	7.9 ± 2.2	7.9 ± 2.4	<0.001
HbA1c, %	7.5 ± 1.5	7.7 ± 1.4	7.7 ± 1.4	<0.001
Total cholesterol, mmol/L	4.75 ± 1.07	4.87 ± 1.14	4.79 ± 1.06	0.553
Triglycerides, mmol/L	1.29 ± 0.74	1.92 ± 1.54	2.11 ± 1.63	<0.001
HDL-C, mmol/L	1.21 ± 0.28	1.10 ± 0.22	1.04 ± 0.22	<0.001
LDL-C, mmol/L	2.95 ± 0.81	3.04 ± 0.80	3.00 ± 0.76	0.337
eGFR, ml/min/1.73 m^2^	88.16 ± 17.55	90.98 ± 16.88	89.32 ± 17.12	0.293
Hypertension, %	69.6	68.2	89.2	<0.001
Dyslipidemia, %	49.8	61.0	74.7	<0.001
Metformin in use, %	25.8	31.0	36.9	<0.001
Thiazolidinediones in use, %	3.7	2.1	2.8	0.274
**POSTMENOPAUSAL WOMEN**				
*N*	637	189	1392	
Age, year	68.9 ± 8.5	65.8 ± 7.3	67.4 ± 7.3	<0.001
Duration of diabetes, year	10.9 ± 8.3	9.3 ± 7.5	9.9 ± 7.5	0.147
Current smoking, %	1.7	2.6	2.2	0.509
AST/ALT	1.35 ± 0.41	1.19 ± 0.35	1.14 ± 0.38	<0.001
Waist circumference, cm	82.3 ± 8.2	80.3 ± 7.9	92.4 ± 8.6	<0.001
FPG, mmol/L	7.4 ± 2.5	7.5 ± 2.4	7.9 ± 2.5	<0.001
HbA1c, %	7.2 ± 1.4	7.4 ± 1.3	7.5 ± 1.3	<0.001
Total cholesterol, mmol/L	5.25 ± 1.21	5.65 ± 1.17	5.37 ± 1.21	0.080
Triglycerides, mmol/L	1.42 ± 0.84	1.87 ± 1.01	2.18 ± 1.67	<0.001
HDL-C, mmol/L	1.43 ± 0.34	1.34 ± 0.28	1.22 ± 0.25	<0.001
LDL-C, mmol/L	3.17 ± 0.89	3.50 ± 0.86	3.32 ± 0.86	0.003
eGFR, ml/min/1.73 m^2^	90.75 ± 16.82	95.16 ± 15.48	92.05 ± 16.15	0.187
Hypertension, %	72.8	60.8	85.3	<0.001
Dyslipidemia, %	47.9	56.1	67.0	<0.001
Metformin in use, %	25.0	32.8	38.3	0.003
Thiazolidinediones in use, %	3.5	2.6	3.6	0.572

*The data are summarized as the mean ± SD for continuous variables or as a numerical proportion for categorical variables. P for trend was calculated by regression tests*.

*In subjects with NAFLD, those who had MetS were categorized into the probable NASH group, and the remaining subjects were placed into the simple NAFLD group*.

*ALT, alanine aminotransferase; AST, aspartate aminotransferase; CTX, collagen type 1 C-telopeptide; eGFR, estimated glomerular filtration rate; FPG, fasting plasma glucose; HDL-C, high-density lipoprotein; HbA1c, glycated hemoglobin; LDL-C, low-density lipoprotein; NAFLD, nonalcoholic fatty liver disease; NASH, nonalcoholic steatohepatitis; P1NP, procollagen type 1 N-terminal propeptide*.

**Table 2:** Characteristics of the participants with NAFLD sorted by fibrosis score.

**Characteristic**	**Probable absence of significant fibrosis** **(<-1.455)**	**Indeterminate result (−1.455, 0.676)**	**Probable presence of significant fibrosis (>0.676)**	***P* for trend**
**NAFLD FIBROSIS SCORE IN MEN**				
*N*	98	874	226	
Age, year	58.2 ± 8.1	66.6 ± 7.2	74.4 ± 7.4	<0.001
Duration of diabetes, year	8.1 ± 6.0	9.4 ± 7.1	10.9 ± 7.6	0.018
Current smoking, %	49.0	35.1	18.6	<0.001
AST/ALT	0.87 ± 0.23	1.02 ± 0.29	1.35 ± 0.67	<0.001
Waist circumference, cm	92.6 ± 7.7	94.1 ± 7.8	98.2 ± 10.1	<0.001
FPG, mmol/L	8.4 ± 2.4	7.9 ± 2.3	7.7 ± 2.4	0.033
HbA1c, %	8.0 ± 1.6	7.7 ± 1.4	7.5 ± 1.3	0.004
Total cholesterol, mmol/L	5.27 ± 1.16	4.84 ± 1.08	4.52 ± 1.02	<0.001
Triglycerides, mmol/L	2.22 ± 1.62	2.13 ± 1.70	1.71 ± 1.14	<0.001
HDL-C, mmol/L	1.08 ± 0.20	1.05 ± 0.22	1.07 ± 0.23	0.707
LDL-C, mmol/L	3.34 ± 0.82	3.02 ± 0.76	2.81 ± 0.77	<0.001
eGFR, ml/min/1.73m^2^	101.60 ± 15.04	90.73 ± 16.18	81.03 ± 17.18	<0.001
Hypertension, %	72.4	83.2	88.5	0.001
Dyslipidemia, %	69.4	73.2	62.4	0.027
Metformin in use, %	40.8	36.3	28.8	0.016
Thiazolidinediones in use, %	2.0	1.9	5.3	0.665
**NAFLD FIBROSIS SCORE IN POSTMENOPAUSAL WOMEN**				
*N*	214	1117	250	
Age, year	61.8 ± 5.4	66.8 ± 6.5	73.9 ± 7.5	<0.001
Duration of diabetes, year	8.6 ± 6.7	9.8 ± 7.5	11.0 ± 7.7	0.002
Current smoking, %	3.7	2.2	1.2	0.070
AST/ALT	0.97 ± 0.25	1.12 ± 0.30	1.45 ± 0.55	<0.001
Waist circumference, cm	87.0 ± 9.2	90.5 ± 8.7	96.2 ± 10.1	<0.001
FPG, mmol/L	9.3 ± 2.6	7.8 ± 2.4	7.7 ± 2.4	0.005
HbA1c, %	7.8 ± 1.4	7.5 ± 1.3	7.4 ± 1.2	0.004
Total cholesterol, mmol/L	5.71 ± 1.18	5.40 ± 1.22	5.18 ± 1.13	<0.001
Triglycerides, mmol/L	2.21 ± 1.22	2.15 ± 1.74	2.04 ± 1.26	<0.001
HDL-C, mmol/L	1.26 ± 0.24	1.24 ± 0.26	1.20 ± 0.27	0.009
LDL-C, mmol/L	3.55 ± 0.85	3.33 ± 0.87	3.19 ± 0.84	<0.001
eGFR, ml/min/1.73m^2^	100.04 ± 13.01	93.13 ± 15.21	82.71 ± 17.79	<0.001
Hypertension, %	76.6	82.0	89.2	<0.001
Dyslipidemia, %	74.3	64.2	64.8	0.044
Metformin in use, %	45.3	37.8	30.4	0.001
Thiazolidinediones in use, %	1.9	3.0	6.8	0.663

*The data are summarized as the mean ± SD for continuous variables or as a numerical proportion for categorical variables. P for trend was calculated by regression tests*.

The NAFLD fibrosis score (NFS) was used to identify NAFLD patients with a higher likelihood of having bridging fibrosis (stage 3) or cirrhosis (stage 4). The following formula was used to calculate the NFS: −1.675 + 0.037 * age (years) + 0.094 * body mass index (kg/m^2^) + 1.13 * IFG/diabetes (yes = 1, no = 0) + 0.99 * AST/ALT ratio – 0.013 * platelet (*10^9^/l) – 0.66 * albumin (g/dl).

*ALT, alanine aminotransferase; AST, aspartate aminotransferase; CTX, collagen type 1 C-telopeptide; eGFR, estimated glomerular filtration rate; FPG, fasting plasma glucose; HDL-C, high-density lipoprotein; HbA1c, glycated hemoglobin; LDL-C, low-density lipoprotein; NAFLD, nonalcoholic fatty liver disease; NASH, nonalcoholic steatohepatitis; P1NP, procollagen type 1 N-terminal propeptide*.

**Figure 1:**
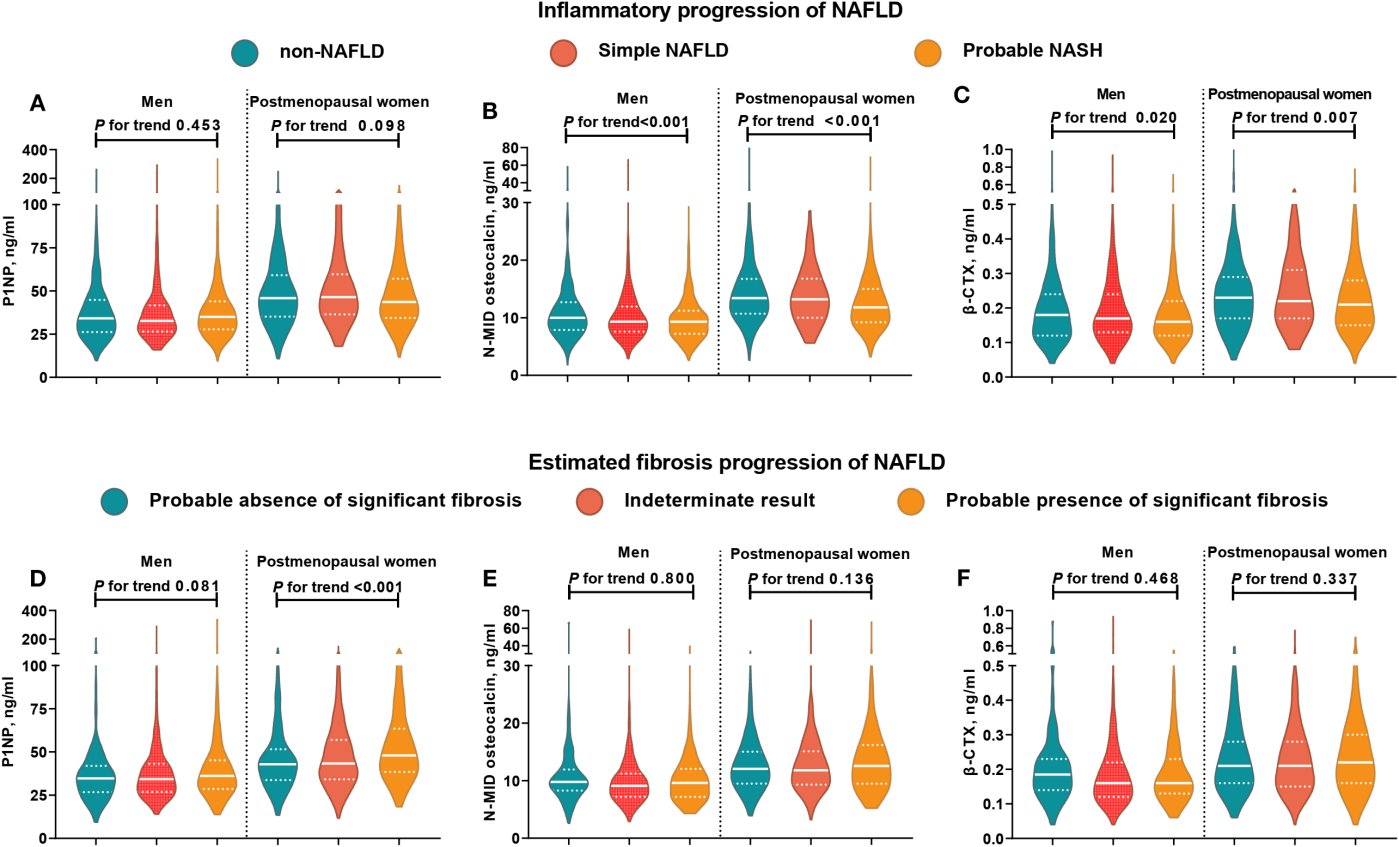
Distribution of bone turnover markers among men and postmenopausal women in different NAFLD categories. In subjects with NAFLD, those who had MetS were categorized into the probable NASH group, and the remaining subjects were placed into the simple NAFLD group. NFS < −1.455 indicated a likely absence of significant fibrosis, NFS > 0.676 indicated the likely presence of significant fibrosis, and NFS between −1.455 and 0.676 indicated indeterminate results. The white solid line represents the median, and the white dotted line represents the lower and upper quartiles. CTX, collagen type 1 C-telopeptide; MetS, metabolic syndrome; NAFLD, nonalcoholic fatty liver disease; NASH, nonalcoholic steatohepatitis; P1NP, procollagen type 1 N-terminal propeptide. **(A–C)** Distribution of bone turnover markers among men and postmenopausal women in different inflammatory progression of NAFLD. **(D–F)** Distribution of bone turnover markers among men and postmenopausal women in different estimated fibrosis progression of NAFLD.

The violin figure shown in Figure 1 presents the distribution of bone turnover markers. Generally, postmenopausal women had higher turnover marker levels than men. From non-NAFLD to simple NAFLD and probable NASH, both men and women had a decreasing trend in N-MID osteocalcin and β-CTX levels, with the P1NP being an exception. However, in the estimated fibrosis progression, none of the turnover markers in men showed significant trend changes, and in women only the P1NP had a significant increasing trend.

### Association Between Bone Turnover Markers and NAFLD in Diabetic Patients

**Figure 2:**
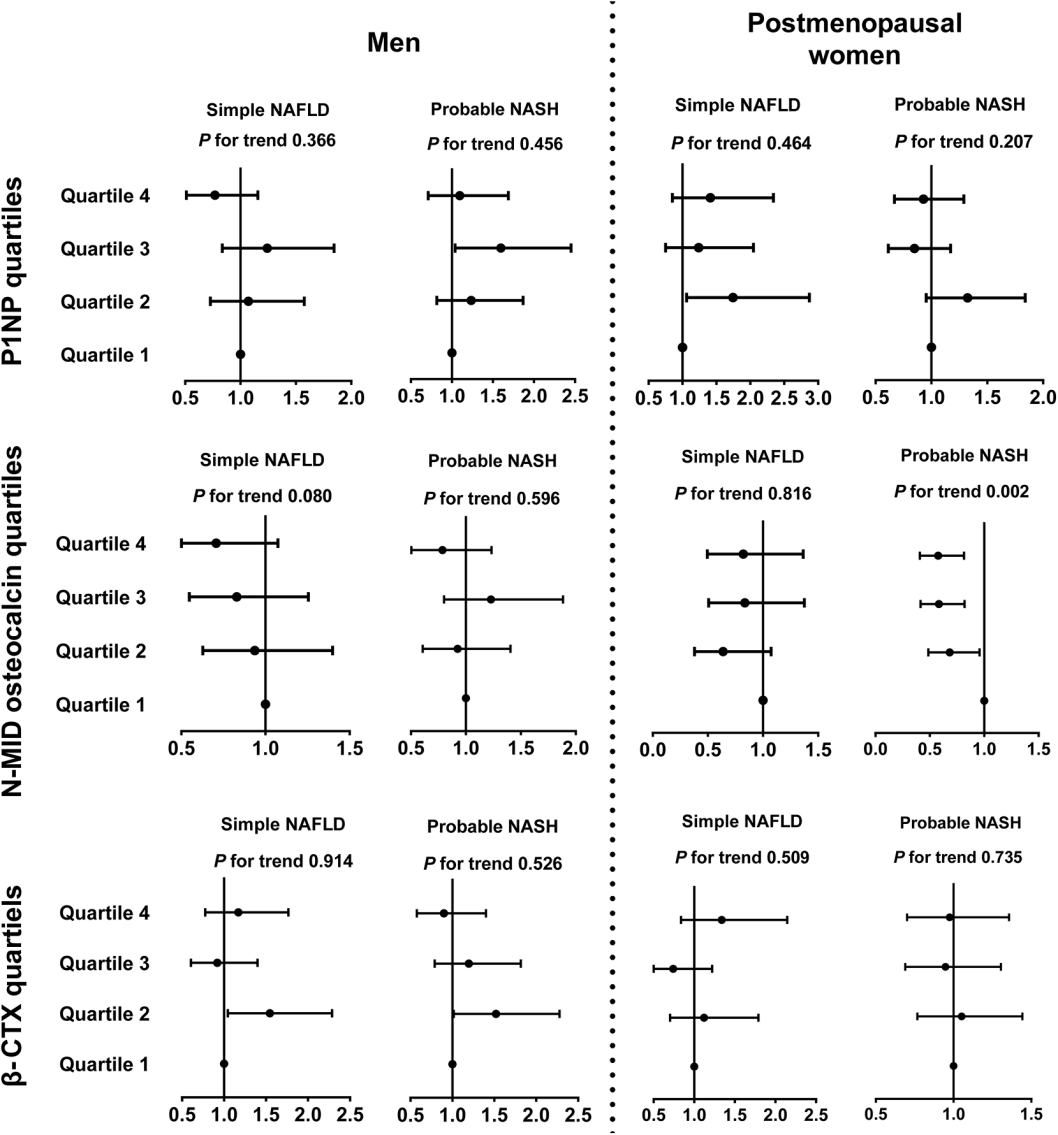
Associations between bone turnover markers and inflammatory progression of NAFLD in diabetic patients. The data are expressed as odds ratios (95% confidence interval). Multinomial logistic regression analysis was used. In subjects with NAFLD, those who had MetS were categorized into the probable NASH group, and the remaining subjects were placed in the simple NAFLD group. The model was adjusted for age, duration of diabetes, HbA1c, current smoking, waist circumference, eGFR, dyslipidemia, hypertension, and use of metformin or thiazolidinediones. CTX, collagen type 1 C-telopeptide; MetS, metabolic syndrome; NAFLD, nonalcoholic fatty liver disease; NASH, nonalcoholic steatohepatitis; P1NP, procollagen type 1 N-terminal propeptide.

After adjusting for demographic and metabolic parameters and diabetes medications in men, we found that none of the P1NP, N-MID osteocalcin, and β-CTX quartiles showed a significant trend associated with simple NAFLD and probable NASH, but some of the quartiles, such as the Q3 of P1NP and the Q2 of β-CTX, were significantly associated with probable NASH compared with the corresponding Q1 (Figure 2).

In postmenopausal women, interestingly, an increase in the N-MID osteocalcin quartile was negatively associated with probable NASH (Q4 vs. Q1, OR 0.58, 95%CI 0.41, 0.82, *P for trend* = 0.002) but not with simple NAFLD. The 1SD increment of ln(N-MID osteocalcin) was also associated with probable NASH (OR 0.57, 95%CI 0.41, 0.79). P1NP and β-CTX quartiles showed no significant association with the inflammatory NAFLD categories (Figure 2).

### Association Between Bone Turnover Markers and Probable Significant Fibrosis in Diabetic Patients With NAFLD

**Figure 3:**
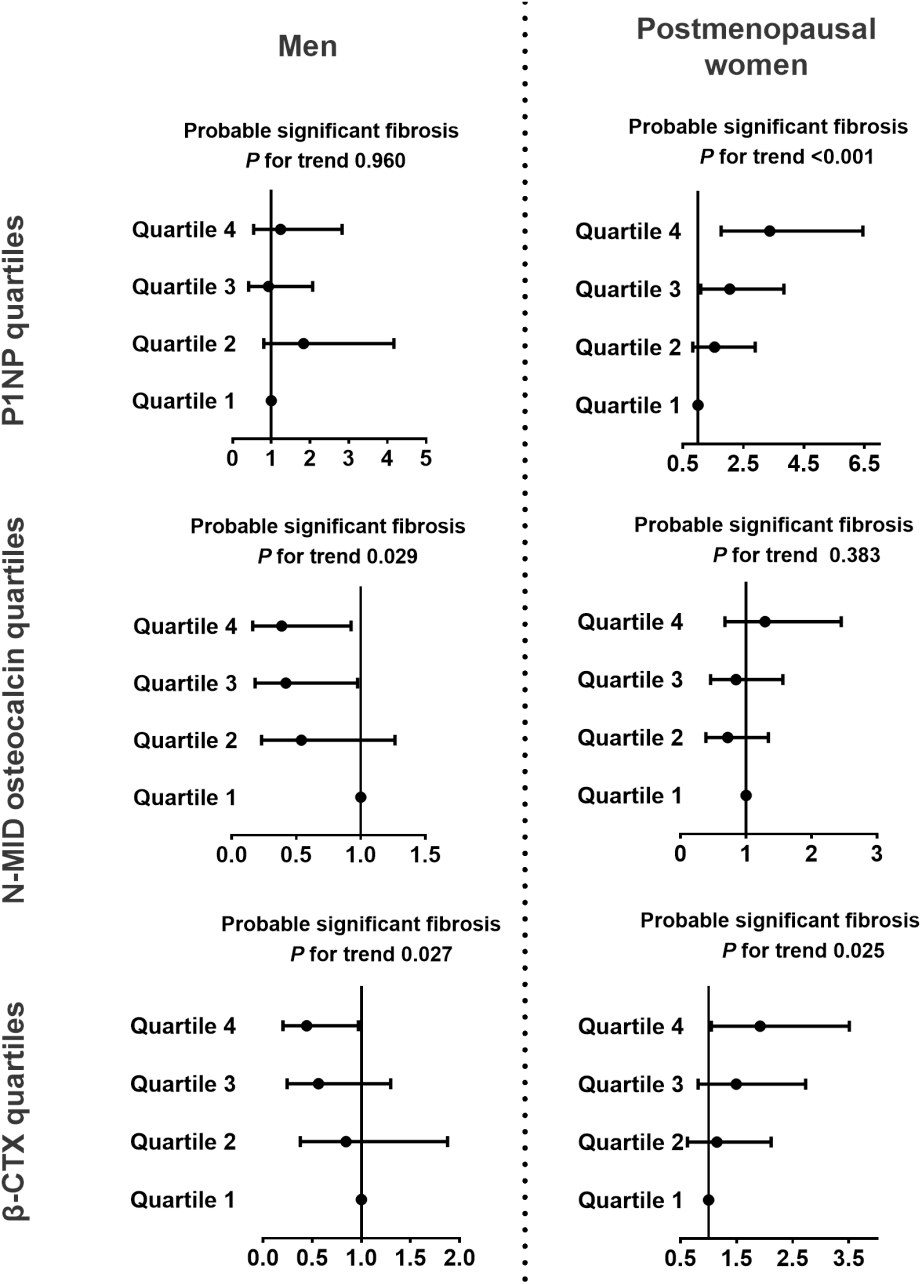
Associations between bone turnover markers and probable significant fibrosis in diabetic patients with NAFLD. NFS <-1.455 indicated a likely absence of significant fibrosis, NFS >0.676 indicated the likely presence of significant fibrosis, and NFS between −1.455 and 0.676 indicated indeterminate results. The data are expressed as odds ratios (95% confidence interval). Multinomial logistic regression analysis was used. The model was adjusted for age, duration of diabetes, HbA1c, current smoking, waist circumference, eGFR, dyslipidemia, hypertension, and use of metformin or thiazolidinediones. CTX, collagen type 1 C-telopeptide; NAFLD, nonalcoholic fatty liver disease; NASH, nonalcoholic steatohepatitis; P1NP, procollagen type 1 N-terminal propeptide.

In postmenopausal women, the P1NP (Q4 vs. Q1, OR 3.37, 95% CI 1.76, 6.44, *P for trend* < 0.001) and β-CTX (Q4 vs. Q1, OR 1.92, 95% CI 1.05, 3.51, *P for trend* 0.025) quartiles were positively associated with the probable presence of significant fibrosis but not with the indeterminate results (Figure 3, Supplementary Figure 2). Among the bone turnover markers, the 1SD increment of ln(P1NP) had the greatest association with the probable presence of significant fibrosis in women (OR 3.83, 95% CI 2.15, 6.83). In men, N-MID osteocalcin and β-CTX quartiles were significantly associated with the group with indeterminate results and probable fibrosis (*P for trend* 0.004, 0.004, 0.029, and 0.027, respectively, Figure 3, Supplementary Figure 2).

## Discussion

In this study, which included 3,982 Chinese diabetic adults (1,764 men and 2,218 postmenopausal women), we report that N-MID osteocalcin is negatively associated with probable NASH, and β-CTX and P1NP are positively associated with the probable presence of significant fibrosis in postmenopausal women. However, in men, N-MID osteocalcin and β-CTX were negatively associated with indeterminate results and with the probable presence of significant fibrosis. Our results indicate that bone turnover might be significantly related to advanced stages of NAFLD in type 2 diabetes. Further understanding of the relationship between bone turnover and liver tissue may help personalize treatment strategies for NAFLD and osteoporosis in patients with type 2 diabetes.

In this study population, we found that N-MID osteocalcin and β-CTX tended to be lower during the inflammatory progression of NAFLD in both men and postmenopausal women. These trends were inconsistent with the negative association between these two bone turnover biomarkers and chronic liver inflammation progression (11, 12, 26, 27). For P1NP in different NAFLD fibrotic progression subgroups, we found that P1NP tended to be higher in postmenopausal women with the progression of fibrosis; however, the levels of P1NP were comparable in men. The possible reason for this gender difference might be that there was a negative and linear relationship between PINP levels and age in men, but a complex relationship was seen in women (28), and age was closely related to the progression of NAFLD fibrosis (Table 1). Moreover, an association between P1NP and probable fibrosis was also found in women but not in men (Figure 3), which supports the finding that P1NP was comparable between different fibrosis risk groups in men.

P1NP has a close relationship with liver fibrosis, both in our study and in other studies. In this study, we found that female participants in the highest P1NP quartile had 3.65-fold higher odds of the probable presence of significant fibrosis after adjusting for age, duration of diabetes, HbA1c, current smoking, waist circumference, eGFR, dyslipidemia, hypertension, and use of metformin or thiazolidinediones. In extremely obese patients with an average body mass index of 44 kg/m^2^, liver biopsy confirmed that the increased stages of fibrosis were independently associated with higher levels of P1NP (29). The P1NP has also been reported to be related to liver fibrosis in other chronic hepatic diseases (10, 30, 31). In primary biliary cirrhosis patients, P1NP levels were higher than in controls (30). In alcoholic cirrhosis patients, the mean serum P1NP concentration was significantly higher than in the control group and appeared to vary depending on the severity of the liver damage (i.e., the concentration of P1NP was significantly higher in Child-Pugh class C than in class A) (10). The concentration of P1NP in the femoral artery blood was also significantly higher in patients with alcoholic cirrhosis than in patients with normal liver function (31).

A possible explanation for the strong association between P1NP and probable significant fibrosis in patients with diabetes and NAFLD is the diverse sources of P1NP, which can originate from the bone or from the liver. P1NP is commonly used as a marker of bone formation because it can be synthesized and secreted by osteoblasts (32). Furthermore, P1NP can also be secreted by hepatic stellate cells and detected in the arterial and hepatic or venous blood (31). In the healthy human liver, the most abundant collagens are the fibril-forming types I and III. During fibrogenesis, type I collagen levels increase up to 8-fold, and the collagen is integrated into the ECM, resulting in fibrosis (33). Since bone and fibrotic livers are both major sources of type I collagen turnover, sources of the P1NP should be distinguished when trying to determine the relative contribution of the bone and the liver to systemic levels of the circulating P1NP. In mature rats subjected to bile duct ligation to construct a liver fibrosis model, the P1NP was specific for the hepatic injury because the bone-specific marker osteocalcin did not increase in the bile duct ligated rats or in the sham-operated rats (33).

In contrast, osteocalcin, which is one of the major noncollagenous proteins in the bone, is more bone specific (32). Although few studies have touched upon N-MID osteocalcin and NAFLD, the possible role of osteocalcin, a less stable form, in NAFLD has been studied. Several cross-sectional studies have suggested that circulating osteocalcin is negatively associated with ALT, aspartate transaminase, fatty liver index and NAFLD (11, 34–37). Osteocalcin is also an independent predictor of the degree of hepatocyte ballooning in NAFLD patients (11, 26, 27). In a weight loss study, an observed increase in circulating osteocalcin was significantly higher in subjects with the highest decrease in ALT levels (34). Moreover, in addition to hepatic steatosis, our study also suggested that a lower circulating osteocalcin level was associated with probable NASH. For liver fibrosis, although osteocalcin was reported to be related to biopsy-confirmed liver fibrosis, the association was not consistently significant in different multivariate regression models examined in different studies (29, 38). According to Luger et al., increased stages of fibrosis are primarily associated with lower osteocalcin and male sex in morbidly obese patients (29). After adjusting for age, sex, BMI, fat mass, and insulin, osteocalcin concentrations were not related to portal inflammation, high grade of steatosis, fibrosis, or lobular inflammation (38). Although metabolic parameters were considered, neither of the studies mentioned kidney function in the regression model (29, 38). In this study, we found that after further adjusting for eGFR, N-MID osteocalcin was negatively associated with probable fibrosis in men but not in postmenopausal women, suggesting the role of gender discrepancy and kidney function in the association between osteocalcin and liver fibrosis.

The association between osteocalcin and probable NASH has some biological plausibility. Evidence suggests that osteocalcin regulates energy homeostasis through multiple pathways (39, 40). An injection with a supraphysiological dose of osteocalcin can reduce fat mass and prevent liver steatosis in wild-type mice fed a high-fat diet (41). Osteocalcin can improve NAFLD by activating the Nrf2 pathway to alleviate oxidative stress and inhibiting the JNK pathway (39). Gprc6a, a putative osteocalcin receptor, mediates its target, molecular T cell factor 7, which modulates Gprc6a and Ucp1 promoter activation and regulates thermogenesis of brown fat (40). Moreover, osteocalcin treatment substantially reduced multiple NASH components with robustly reduced expression of proinflammatory and profibrotic genes in the liver and proinflammatory genes in the white adipose tissue (42).

Another bone turnover marker, β-CTX, which represents type 1 collagen, may also be associated with chronic liver disease progression. For inflammatory progression of chronic liver disease, the CTX level in alcoholic liver disease patients was found to be significantly lower than in healthy controls and was negatively correlated with proinflammatory cytokines but positively correlated with anti-inflammatory cytokines (12). For fibrotic progression of chronic liver disease, in this study, we found that CTX was independently associated with the probable presence of fibrosis with an opposite tendency in men and postmenopausal women after adjusting for confounders, although its level was not significantly different in patients with diverse fibrosis risk stratification. Gender discrepancy in the CTX levels exists throughout all age groups (28, 43). In women, the β-CTX levels are increased after the age of 50. Before the age of 50, the β-CTX level in men is higher than that in women, but after the age of 50, the reverse trend is evident (28). The different trends in CTX alteration in men and women after the age of 50 might provide a possible explanation for the different tendencies in the association between β-CTX and probable fibrosis. However, because in men β-CTX was significantly associated with both an indeterminate and probable presence of liver fibrosis, its discrimination value should be further evaluated.

Although our study has some strengths, including a relatively large sample size of community dwelling participants and strong quality control, there were also some limitations. First, this study is a cross-sectional study, and thus, it cannot identify a causal relationship between bone turnover markers and NAFLD progression. Second, the study population was from a single center, and this could introduce a selection bias. Third, we used steatosis on ultrasound with MetS to define probable NASH and used the NFS to evaluate fibrosis in patients with type 2 diabetes. Although liver histology is the most accurate method to assess the liver histological progression stages, this invasive examination could not be applied in this large-scale epidemiology study. Because MetS is a strong predictor of the presence of NASH in patients with NAFLD (23), we used it for risk stratification of NAFLD inflammatory progression. The NFS can accurately predict the presence or absence of advanced fibrosis in NAFLD and was suggested by the American Association for the Study of Liver Diseases in 2018 to be used as a clinical decision aid to identify patients with type 2 diabetes suspected of having NAFLD or NASH (23). Fourth, hepatic steatosis may decrease and eventually disappear as fibrosis progresses, and thus, the absence of steatosis might include the presence of advanced fibrosis. Fifth, collagen generation is a key factor in liver fibrosis progression, and changes in collagen biomarker levels might coexist with both bone turnover and liver fibrogenic processes. In this study, we could not distinguish the source of the P1NP or β-CTX. Last, bone metabolism status could be more comprehensively described with both bone turnover markers and bone mineral density (BMD). However, BMD was not collected in the study population. Future studies involving BMD are called for to better understand these association.

In conclusion, significant associations among N-MID osteocalcin, β-CTX and P1NP, and probable advanced NAFLD were observed. Further prospective and animal studies are warranted to understand the causal relationship and underlying mechanism.

## Data Availability Statement

The datasets generated for this study are available on request to the corresponding author.

## Author Contributions

YL, BH, BJ, and NW contributed to the conception and design of the study. NW, YW, XC, WZ, YC, FX, HW, and QL contributed to acquisition, analysis, and interpretation of data. NW, YW, and XC drafted the article. YL, BH, and BJ critically revised the manuscript for important intellectual content. All authors approved the final version submitted.

### Conflict of Interest

The authors declare that the research was conducted in the absence of any commercial or financial relationships that could be construed as a potential conflict of interest.
