# Supplementary material for: Bone Turnover Markers and Probable Advanced Nonalcoholic Fatty Liver Disease in Middle-Aged and Elderly Men and Postmenopausal Women With Type 2 Diabetes
Source: Front Endocrinol (Lausanne). 2020 Jan 28;10:926. doi: 10.3389/fendo.2019.00926 (PMC6999074; doi:10.3389/fendo.2019.00926)
Supplement: Supplementary file 1 [file Data_Sheet_1.pdf]

Supplementary Material

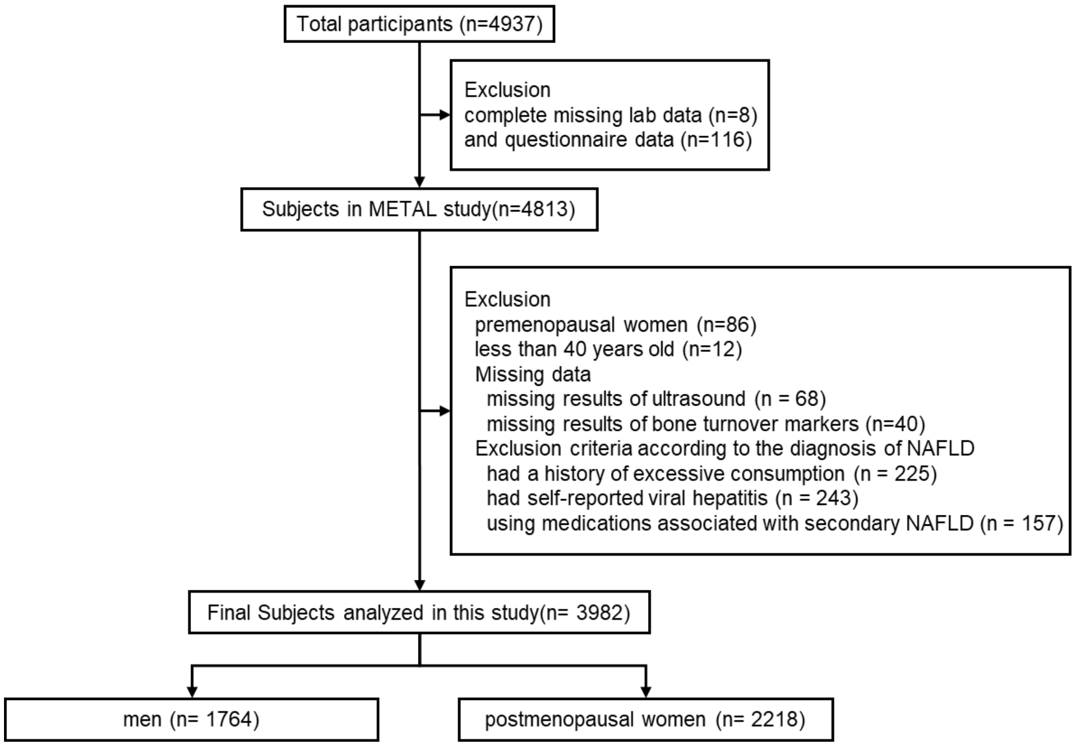

Supplementary Figure 1. Flowchart of participants' inclusion and exclusion

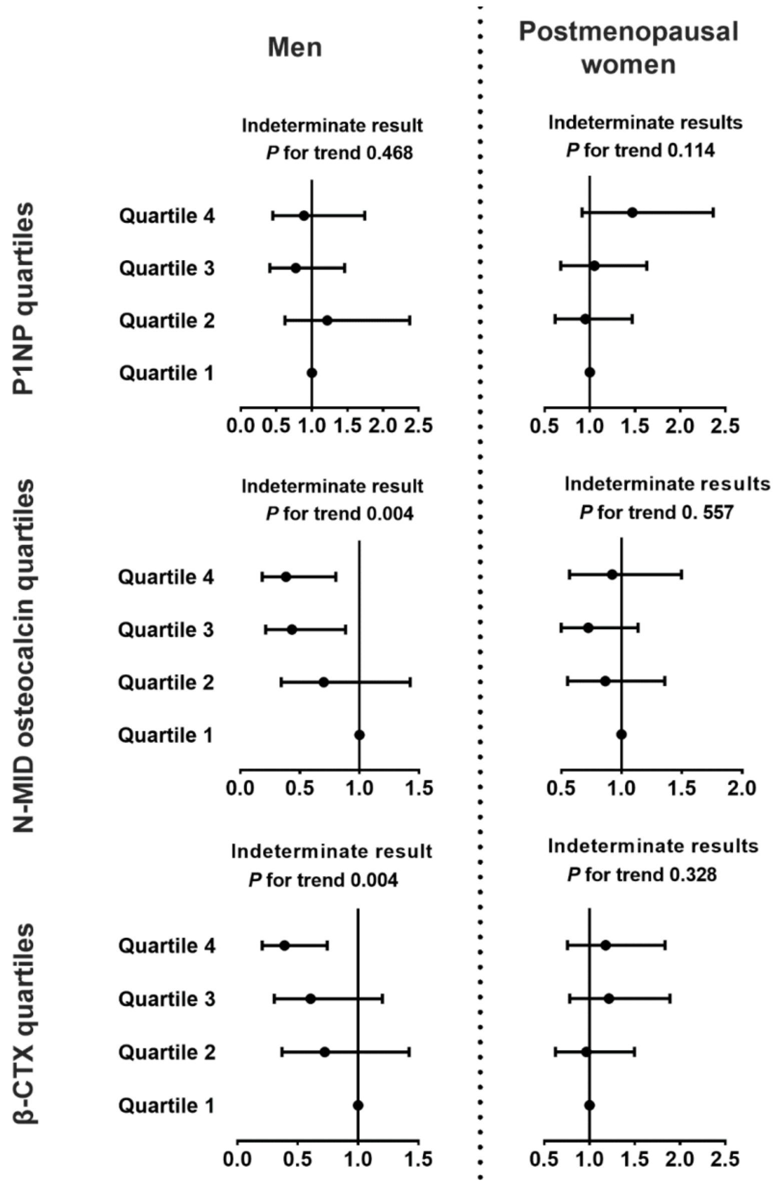

Supplementary Figure 2. Associations of bone turnover markers with indeterminate result of fibrosis in diabetic patients with NAFLD. Data are expressed as odds ratios (95% confidence interval). Multinomial logistic regression analysis was used. CTX, collagen type C-telopeptide; NAFLD, nonalcoholic fatty liver disease; NASH, non-alcoholic steatohepatitis; P1NP, procollagen type 1 N-terminal propeptide. The model was adjusted for age, duration of diabetes, HbA1c, current smoking, waist circumference, dyslipidemia, hypertension, eGFR, metformin in use and thiazolidinediones in use.
